# Supplementary material for: Light storage in light cages: a scalable platform for multiplexed quantum memories
Source: Light Sci Appl. 2026 Jan 1;15:13. doi: 10.1038/s41377-025-02085-5 (PMC12756318; doi:10.1038/s41377-025-02085-5)
Supplement: Supplementary file 1 — Supplementary Information for: Light Storage in Light Cages: A Scalable Platform for Multiplexed Quantum Memories [file 41377_2025_2085_MOESM1_ESM.pdf]

Supplementary Information for: Light Storage in Light Cages: A Scalable Platform for Multiplexed Quantum Memories

Esteban Gómez-López[1*], Dominik Ritter[1], Jisoo Kim[2], Harald Kübler[3], Markus A. Schmidt[2,4], Oliver Benson[1]

[1*]Department of Physics, Humboldt-Universität zu Berlin, Berlin, 12489, Germany.
[2]Department of Fiber Photonics, Leibniz Institute of Photonic Technology, Jena, 07745, Germany.
[3]5th Institute of Physics, University of Stuttgart, Stuttgart, 70569, Germany.
[4]Otto Schott Institute of Material Research, Jena, 07743, Germany.

*Corresponding author(s). E-mail(s): egomez@physik.hu-berlin.de;

S1 Light cage: Optical properties

The key properties relevant to our current study have been documented in several prior works [1–3]. Considering this extensive body of literature and the practical limitations of our current sample, which is embedded in a cesium vapor cell and unavailable for further broadband characterization measurements, we find it appropriate to summarize the main properties here by referencing the literature.

The light cages guide light through the formation of core modes caused by the antiresonance effect resulting from the reflection properties of the microstructured strands forming the cladding. This creates leaky modes that dissipate energy along the propagation axis [1]. The modal attenuation, α, i.e., the energy loss of the core mode per millimeter, is a key parameter that determines how well the light cages can serve as waveguides to boost light-matter interaction. In fact, in Ref. [2], it has been shown that the light cages enhance light-matter interaction by increasing the Rabi frequency in continuous wave electromagnetically induced transparency. Meanwhile, the broad side-wise access to the core allows atomic vapor to quickly diffuse into the core, dramatically reducing filling times compared to months in other hollow-core

waveguides. Despite these advantages, the modal attenuation was relatively large, $\alpha = 5$ dB mm^{-1}, which limits the Rabi frequency achievable inside the LC.

In table 1 we summarize the properties of the light cage system used in our work compared to Ref. [3], where a waveguide printed with the same nanoprinting strategy was investigated, and therefore their optical properties are expected to be similar. Indeed, the measured off-resonance losses at 900 nm, 1.0 - 1.5 dB mm^{-1}, are comparable to our system enclosed inside the Cs vapor cell, α = 1.19(3) dB mm^{-1} at 894 nm.

**Property**	**This Study**	**Ref. [3]**
Number of strands	12	12
Strand diameter	3.6 µm	3.6 µm
Inter-strand distance (pitch)	7 µm	7 µm
Core diameter	28 µm	28 µm
Sample length	4.5 mm	up to 30 mm
Aspect ratio (length/diameter)	1250	up to 8200
Measured losses (off-resonance, 900 nm)	not measured	1.0–1.5 dB/mm
Intrachip strand diameter variation	not measured	2 nm (average)
Interchip strand diameter variation	not measured	15 nm (average)
Polarization sensitivity	negligible	negligible

Table 1 Overview of key properties of the light cage structure investigated in this work (center column) compared to benchmark values reported in the literature (right column).

S2 Transmission efficiency

To measure the transmission efficiency of the investigated light cages, we utilized a signal laser field red detuned by 4 GHz from the Cs D1 hyperfine state $F = 3 - F' = 3$. At this detuning, the optical depth of the atomic vapor is $< 10^{-3}$ at 74 °C, rendering it negligible for the transmission efficiency measurements. The input power is recorded before the coupling lens (L1) at position $\mathrm{P_{in}}$, while the output power is measured behind an iris located at the output of the collimation lens (L2), position $\mathrm{P_{out}}$, as shown in Fig. S2a. The iris filters out stray light from the signal laser that did not couple into the waveguide. Therefore, the measured transmission efficiency, $\eta_T = P_{\mathrm{out}}/P_{\mathrm{in}}$, corresponds only to the coupled mode into the LC. The resulting efficiencies are $\eta_{T,A} = 0.20(1)$ for the LC-A and $\eta_{T,B} = 0.14(1)$ for LC-B. Additionally, the transmission of the vapor cell without the LC was recorded by displacing the LC out of the optical path and refocusing the beam spot into the middle of the vapor cell, as indicated in Fig. S2b. The measured transmission under the same temperature and detuning is $\eta_{\mathrm{cell}} = 0.82(1)$. This value includes the reflections on lenses L1 and L2 as well as the windows of the vapor cell.

The in-coupling efficiency into the light cages, η_{coup}, is given by the modified Beer-Lambert law [4]:

$$P = P_0\eta_T \times 10^{-(1-f)\epsilon c L_{\mathrm{cell}} - (\alpha/10\mathrm{dB})L_{\mathrm{LC}}}, \tag{1}$$

with $\eta_T = \eta_{\text{cell}}\eta_{\text{coup}}$, P the output power, P_0 the input power, ϵ the molar attenuation coefficient, c the molar concentration of Cs, L_{cell} the vapor cell length, L_{LC} the light cage length, α the modal attenuation (in dB m^{-1}), and f the fraction of power inside the strands. We can simplify the expression knowing that f for the used light cages has been calculated to be negligibly small ($f < 10^{-3}$)[4]. Taking into account as well the small optical density when using off-resonant light, $\epsilon c L_{\text{cell}} < 10^{-3}$, the power reduction at the output can be well approximated by the equation

$$P = P_0 \eta_{\text{cell}} \eta_{\text{coup}} \times 10^{-(\alpha/10\text{dB})L_{\text{LC}}} \tag{2}$$

The modal attenuation of the LCs, measured before placing the chip inside the vapor cell, is $\alpha = 1.19(3)$ dB mm^{-1} for LC-A and $\alpha = 1.34(5)$ dB mm^{-1} for LC-B. Using these values, we can estimate the coupling efficiency into the LCs, resulting in $\eta_{coup,A} = 0.82(4)$ and $\eta_{coup,B} = 0.68(6)$, respectively. This highlights that in-coupling from outside of the vapor cells can reach acceptable high values. To increase the overall transmission efficiencies, improved printing techniques can be used to reduce the modal attenuation, as well as direct fiber coupling to increase the in-coupling efficiency above 90%.

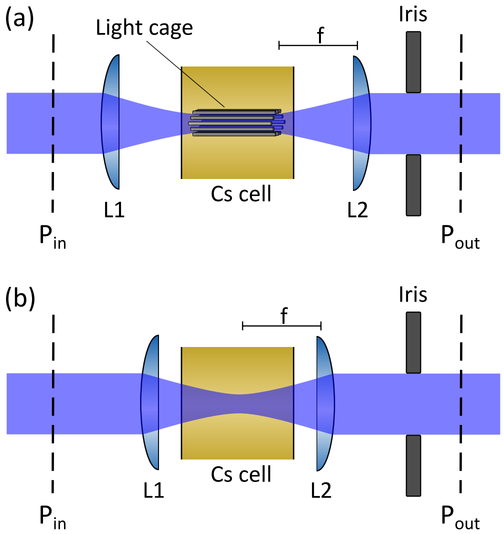

Fig. S1 Transmission efficiency measurement. (a) Measurement of the transmission efficiency through the light cage. The power of the signal laser field is measured at positions P_{in} and P_{out}. An iris is used to block stray light that did not couple into the waveguide. The laser was red detuned 4 GHz from the transition $F = 3 - F' = 3$ to reduce the atomic absorption below 0.001 OD at 74 °C. The resulting transmission efficiencies of the investigated LCs are $\eta_{T,A} = 0.21(1)$, $\eta_{T,B} = 0.13(1)$. **(b)** Measurement of the transmission efficiency only through the vapor cell, $\eta_{\text{cell}} = 0.82(1)$ is recorded under the same detuning conditions.

S3 Electromagnetically induced transparency in lossy waveguides

The spectra measured of light coupled into a light cage show spectral features absent in a focused free space scenario (see main text Fig. 3). These additional “shoulder-like” features can be explained considering an exponentially decaying Rabi frequency of the control field $\Omega_c(z)$, as treated in Ref. [2]. The maximum Rabi frequency Ω_0 is achieved at the focal spot of the lens used to couple the light into the LC. This value decays exponentially as the light propagates through the LC with a modal attenuation α, i.e., $\Omega(z) = \Omega_0\, 10^{-\alpha z/20\mathrm{dB}}$, with a measured $\alpha = 1.19(3)$ dB mm^{-1} in our experiment (see Sec. S2 of this Supplementary Information for details on efficiencies and losses).

The electrical susceptibility of the atomic media under EIT at a specific position z is given by the expression [5]:

$$\chi(\Delta\nu_s, z) = \frac{|\mu_{31}|^2\,\varrho}{\epsilon_0\,\hbar}\left[\frac{4\,\Delta\,(\Omega_c^2(z) - 4\,\Delta^2 - \gamma_d^2)}{|\,\Omega_c^2(z) + (\gamma_{31} + i\,2\,\Delta)(\gamma_d + i\,2\,\Delta)\,|^2} + i\,\frac{8\,\Delta^2\,\gamma_{31} + 2\,\gamma_d\,(\Omega_c^2(z) + \gamma_{31}\,\gamma_d)}{|\,\Omega_c^2(z) + (\gamma_{31} + i\,2\,\Delta)(\gamma_d + i\,2\,\Delta)\,|^2}\right] \tag{3}$$

Then, we calculate the measured transmission spectra of the light coupled into the LC in the alkali atmosphere by integrating the susceptibility along the length L of the vapor cell,

$$T(\Delta\nu_s) = \exp\left[-\frac{4\pi\nu_0}{c_0}\,\mathrm{Im}\left\{\int_0^L \tilde{\chi}(\Delta\nu_s, z)\,dz\right\}\right] \tag{4}$$

where $\tilde{\chi}(\Delta\nu_s, z)$ is the Doppler broadened susceptibility of the atomic ensemble, calculated as the convolution of the susceptibility $\chi(\Delta\nu_s, z)$ with a Maxwell-Boltzmann distribution of the atomic velocities given a mean thermal velocity [6]. It is important to note that the atomic density ϱ also depends on the temperature of the gas in the cell and is calculated following Ref. [7]. The maximum control Rabi frequency Ω_0 is left as a fitting parameter, as well as the temperature, to account for the reduction of atomic density inside the LC.

From the susceptibility $\tilde{\chi}(\Delta\nu_s, z)$, we can compute the dispersion of a light pulse through the atomic media using the refractive index, $n = \mathrm{Re}(1 + \chi)$, in particular, the modification of the group velocity under EIT at the two-photon resonance is [5]:

$$v_g = \left.\frac{c}{n + \nu_s \frac{dn}{d\nu_s}}\right|_{\Delta\nu_s = 0} \tag{5}$$

with c the speed of light in vacuum. From this equation, the spatial compression of a propagating light pulse can be calculated as $L_{\mathrm{EIT}} = L_0\, v_g/c$. Utilizing the linear susceptibilities $\tilde{\chi}(\Delta\nu_s, z)$ obtained from the fitted transmission spectra (main text Fig. 3) we compute the refractive index n, shown in Fig. S2a. At the two-photon resonance ($\Delta\nu_s = 0$), a positive slope that decreases with increasing control power P_c

is observed as expected. The spatial compression of the signal light pulse as a function of P_c is computed using Eq. (5) and displayed in Fig. S2b. Notably, the compressed pulses have a spatial extension considerably larger than the vapor cell, explaining the limited efficiency considering the achieved optical depth, both crucial parameters for optimal storage [8]. Furthermore, increasing P_c increases the memory bandwidth at the cost of spatial compression, reducing the memory efficiency for higher bandwidths, as observed in Fig. 5-(b), -(d). Therefore, an increase in the optical depth is required to achieve greater efficiency at the current bandwidths. Using the achieved experimental parameters we calculate that OD > 7.7, would be required for a signal pulse with 14 ns temporal width to achieve a compression $\Delta L_{\mathrm{EIT}} < 4.5$ mm. Alternatively, printing a LC 10 mm long would reduce the required density to OD > 3.5 to spatially compress the signal pulse within the waveguide, resulting in a reduction of more than 10 °C in the required temperature to achieve this compression.

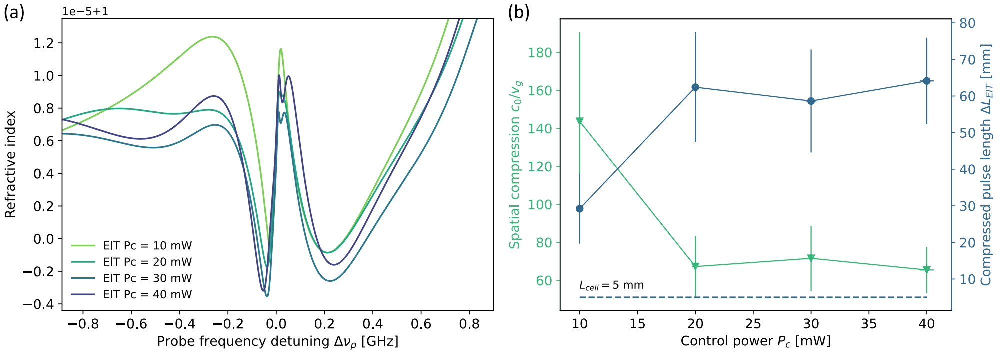

Fig. S2 Spatial pulse compression in the LC (a) Simulated refractive index under the measured EIT conditions in the LC. For increasing control powers (P_c), the steepness of the slope on resonance is reduced. (b) Spatial compression factor c_0/v_g and the compressed length of a pulse after propagation through the LC as a function of P_c, for a signal pulse width $\Delta t_s = 14$ ns. Only the portion of the signal pulse that fits the LC and vapor cell (dashed line) will be stored in the atomic media.

S4 Storage sequence of light pulses

The detected intensities and arrival times for varying set storage times are shown in Fig. S3a. Here, an offset between the measured storage time (t_{storage}) and the set storage time (t_{set}) is visible. This effect arises from the storage sequence utilized in our experiments. The control field consists of a pump pulse followed by two Gaussian pulses of full width at half maximum (FWHM) Δt_c, labeled write and read pulses, as shown in Fig. S3b. The input signal is also a Gaussian pulse with FWHM Δt_s, following a fixed ratio to the width of the control pulses, $\Delta t_s = (2/3)\,\Delta t_c$. The signal is synchronized to arrive with a delay t_{delay} from the center of the write pulse. The highest read intensity is achieved by letting the signal arrive on the latter part of the write pulse. This delay was found to be $t_{\mathrm{delay}} = (1/3)\,\Delta t_c$. Due to the time-reversal condition for optimal retrieval [9], this results in a retrieved pulse arising on the leading

part of the read pulse. The actual storage time, or measured storage time (t_{storage}), is therefore shorter than the set storage time, i.e., $t_{\mathrm{storage}} < t_{\mathrm{set}} - (2/3)\,\Delta t_c$.

In Fig. S3a, the control width is kept constant while the set storage time gradually increases, resulting in a continuous shift between t_{set} and t_{storage}. As an example, we take the measured histogram at $t_{\mathrm{set}} = 90$ ns with a measured storage time of 52.37(4) ns, see Fig. S3b. In the case of constant t_{set} with increasing Δt_s, this discrepancy with the measured storage time becomes more evident as wider control pulses require longer t_{delay}, resulting in progressively shorter storage times, see main text Fig. 5-(c).

Estimation of photon number and signal to noise ratio

To calculate the average photon number per pulse in our experiment, we employ the expression:

$$|\alpha|^2 = \frac{N_{\mathrm{in}}}{f_{\mathrm{rep}}\eta_{\mathrm{e2e}}t_{\mathrm{int}}} \tag{6}$$

with the input field counts N_{in}, the experiment repetition rate $f_{\mathrm{rep}} = 0.5$ MHz, the integration time per storage measurement $t_{\mathrm{int}} = 5$ s and the end-to-end efficiency of the setup $\eta_{\mathrm{e2e}} = 0.0021(1)$. The input counts N_{in} are the integrated counts over a temporal filter of width $3\sigma_{\mathrm{in}}$ around the maximum counts of the input pulse, resulting in an average photon number $|\alpha|^2_{\mathrm{in}} = 50(5)$. Analogous analysis is performed for the noise, where the signal is blocked and only the control pulses are measured, obtaining $|\alpha|^2_{\mathrm{noise}} = 0.02(1)$. From here, a high signal-to-noise ratio, SNR $= 2363$ ratio is deduced. The histogram of the noise is amplified 100-fold and displayed in Fig. S3b (orange trace) for better visibility.

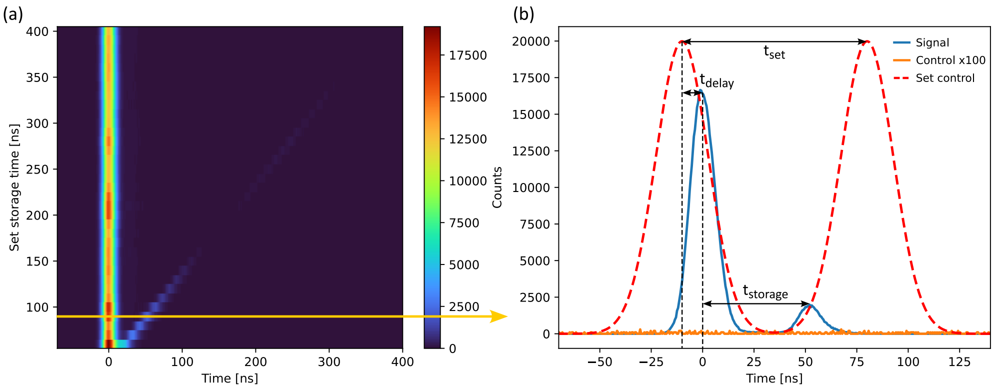

Fig. S3 Set and measured storage times. (a) Heat map of the intensity of the detected signal for varying storage times with $P_c = 10.0(1)$ mW, $\Delta t_s = 14.1(4)$ ns. **(b)** Example of an individual measurement for a set storage time $t_{\mathrm{set}} = 90$ ns. The signal without noise correction comprises the leak and read pulses (blue curve), where the center of the leak is used as the origin of the time scale. The control field intensity (orange curve), here scaled by a factor of 100, evidence the high signal-to-noise ratio achieved. A reconstruction of the control pulses (dashed curve). t_{delay} corresponds to the set delay between the write pulse and input signal, t_{set} is the set time between write and read pulses, t_{storage} the measured storage time.

S5 Magnetic field influence on the storage efficiency

The decrease of the efficiency of a quantum memory as a function of the storage time can be modeled using an exponential with decay constant t_{mem} [10, 11], the so-called memory lifetime. In Fig. 4 and Fig. 5 of the main text, we can see an apparent deviation from the expected exponential decay in the form of a damped oscillation of the storage efficiency.

The reason is that light is stored as a spin-wave excitation in the atomic vapor, which makes the memory sensitive to spin precession caused by stray magnetic fields. These oscillations result in a change to the initially linearly polarized signal light after storage. However, since we employ polarization filtering, a portion of the signal is filtered out and goes undetected. Residual magnetic fields are expected since the vapor cell has no magnetic shielding. To extract t_{mem} from the measured data, a heuristic fit that accounts for the precession is used in the form of an exponentially damped oscillation of the efficiency with a corresponding Larmor frequency of the electronic spins, $\omega = \gamma B$ (with γ the electron gyromagnetic ratio):

$$\eta_{\mathrm{int}} = e^{-t/t_{\mathrm{mem}}} \sin^2\left(\frac{\omega}{2}t + \phi\right) \tag{7}$$

The fit and its 95% confidence interval are shown in Fig. 4-(b) in the main text, by having t_{mem}, ω and an arbitrary phase ϕ as fitting parameters. There is excellent agreement with the experimental data, and we find a memory lifetime of $t_{\mathrm{mem}} = 84(2)$ ns. The spin precession frequency obtained from this model is $f = 3.573(2)$ MHz, corresponding to a magnetic field of 127.49(5) μT. This agrees with the experimental conditions, considering the Earth's magnetic field intensity around 50 μT [12]. In addition, the vapor cell is heated using a pair of resistors with a current of 4 A, inducing an estimated magnetic field of 80 μT in the region of the LC chip. The observed spin precession offers an advantageous way to finely control the polarization of the stored light pulse. Magnetic polarization control of stored light in a quantum memory will thus be a subject of further study.

S6 Dominant decoherence processes in a light cage memory

In our LC memory, the storage lifetime is limited by two dominant decoherence processes: decoherence of spin states due to magnetic fields and time-of-flight broadening. The vapor cell and its oven are not magnetically shielded and therefore the atomic vapor within the LC is affected by stray magnetic fields, as well by the induced fields coming from the resistive heaters of the oven. As discussed in this supplementary information, S5, the magnetic fields produce a Larmor frequency of $f = 3.573(2)$ MHz. Half-period of this oscillation can be considered as the decoherence time in the memory due to magnetic fields, i.e., 140 ns. The implementation of magnetic shielding and active field suppression would reduce the magnetic spin decoherence, boosting the memory lifetime to the microsecond regime [13].

The fast diffusion of atoms into the core of the LC allowed by the side-wise access of the waveguide [2], indicates that the atoms can freely enter and leave the core region

of the waveguide. Given the small diameter of our waveguides, $D = 28\ \mu m$, the time-of-flight (TOF) of the atoms to travel across the core becomes relevant. The mean thermal velocity, v_{th}, of a gas with atomic mass m at temperature T is

$$v_{th} = \sqrt{\frac{8k_B T}{\pi m}} \tag{8}$$

where k_B is the Boltzmann constant. For Cs at 74 °C we have $v_{th} = 235$ m s^{-1}. This implies the TOF for an atom that enters the LC is 119 ns, under the assumption of a transversal trajectory and a large mean free path. This can be considered the decoherence time caused by atoms leaving the LC, thereby reducing the interaction volume. To increase the TOF, the diameter of the LC can be increased, resulting in a linear scaling with the core diameter. Additionally, buffer gases, such as neon, can be used to reduce the diffusion of the Cs atoms inside the LC by increasing the collision of Cs with the buffer gas via spin-preserving collisions, therefore mitigating this decoherence channel [14].

References

[1] Jain, C. *et al.* Hollow Core Light Cage: Trapping Light Behind Bars. *ACS Photonics* **6**, 649–658 (2019).

[2] Davidson-Marquis, F. *et al.* Coherent interaction of atoms with a beam of light confined in a light cage. *Light: Science & Applications* **10**, 114 (2021).

[3] Bürger, J. *et al.* Ultrahigh-aspect-ratio light cages: Fabrication limits and tolerances of free-standing 3D nanoprinted waveguides. *Optical Materials Express* **11**, 1046–1057 (2021).

[4] Kim, J. *et al.* The Optofluidic Light Cage – On-Chip Integrated Spectroscopy Using an Antiresonance Hollow Core Waveguide. *Analytical Chemistry* **93**, 752–760 (2021).

[5] Fleischhauer, M., Imamoglu, A. & Marangos, J. P. Electromagnetically induced transparency: Optics in coherent media. *Reviews of Modern Physics* **77**, 633–673 (2005).

[6] Figueroa, E. *et al.* Decoherence of electromagnetically induced transparency in atomic vapor. *Optics Letters* **31**, 2625–2627 (2006).

[7] Siddons, P. *et al.* Absolute absorption on rubidium D lines: Comparison between theory and experiment. *Journal of Physics B: Atomic, Molecular and Optical Physics* **41**, 155004 (2008).

[8] Gorshkov, A. V., Novikova, I. & Phillips, N. B. Optimal light storage in atomic vapor. *Physical Review A* **78**, 023801 (2008).

[9] Gorshkov, A. V. *et al.* Universal Approach to Optimal Photon Storage in Atomic Media. *Physical Review Letters* **98**, 123601 (2007).

[10] Phillips, D. F. *et al.* Storage of Light in Atomic Vapor. *Physical Review Letters* **86**, 783–786 (2001).

[11] Katz, O. & Firstenberg, O. Light storage for one second in room-temperature alkali vapor. *Nature Communications* **9**, 2074 (2018).

[12] Alken, P. *et al.* International Geomagnetic Reference Field: The thirteenth generation. *Earth, Planets and Space* **73**, 49 (2021).

[13] Ma, L. *et al.* High-performance cavity-enhanced quantum memory with warm atomic cell. *Nature Communications* **13**, 2368 (2022).

[14] Finkelstein, R. *et al.* A practical guide to electromagnetically induced transparency in atomic vapor. *New Journal of Physics* **25**, 035001 (2023).
